# Granger causality is designed to measure effect, not mechanism

**DOI:** 10.3389/fninf.2013.00006

**Published:** 2013-04-25

**Authors:** Adam B. Barrett, Lionel Barnett

**Affiliations:** Department of Informatics, Sackler Centre for Consciousness Science, University of SussexBrighton, UK

In their recent paper, Hu et al. (2011) make the claim that Granger causality (GC) does not capture how strongly one time series influences another. Given the sizeable literature on GC, this claim could be considered radical. We examined this claim, and found that it is based essentially on semantics. Hu et al. (2011) would like a measure of causal interaction to explicitly quantify an underlying causal mechanism, and point out that GC values do not consistently reflect the relative sizes of explicit interaction coefficients in a corresponding generative model. However, GC is, by design and purpose, not interested in this. Rather, it is a measure of causal effect, namely the reduction in prediction error when the causal interaction is taken into account, as compared to when it is ignored. [According to one version of neuroscience terminology (Friston, 2011), which attempts to draw a distinction between the different conceptions of connectivity, GC measures of causal effect yield directed “functional connectivity” maps when applied to neuroimaging data. In contrast, “effective connectivity” maps represent the effective mechanism generating the observed data, and provide interaction coefficients. Neither functional nor effective connectivity representations necessarily map univocally onto the underlying anatomical (structural) connectivity.]

Multiple properties of GC make it an elegant measure of causal effect. It satisfies crucial symmetry properties, including that GC from *Y* to *X* is invariant under rescalings of *Y* and *X*, as well as the addition of a multiple of *X* to *Y*, consistent with the measuring of *independent* predictive information about *X* contained in *Y* (Geweke, 1982; Hosoya, 1991; Barrett et al., 2010). Such transformations do however, change the relative magnitudes of regression coefficients, thus it is not possible to simultaneously measure causal mechanism and causal effect. Further, for the case of Gaussian variables, GC is equivalent to transfer entropy, enabling an explicit interpretation in terms of Shannon information flow (Barnett et al., 2009). The GC from one multivariate variable to another multivariate variable has a decomposition into the sum of independent contributions from each predictor to each predictee [equation 18 in Barrett et al. (2010)]. The same defence of GC applies in the frequency domain, with spectral GC from *Y* to *X* at frequency *f* capturing the proportion of power of *X* at frequency *f* that results from its interaction with *Y* (Geweke, 1982). The fact that time domain GC is the mean spectral GC over all frequencies up to the Nyquist frequency provides further justification.

As far as statistical inference is concerned, for the reasons above, GC can indeed be used to compare the magnitude of causal interactions between different sets of time series. Contrary to Hu et al.'s (2011) interpretation, the fact that some regression coefficients contribute more to GC than others (and some not at all) is actually an indication that GC analysis adds to our understanding of a system, even when the generative model is known *a priori*. A further pragmatic advantage of the GC method is that, in sample, time-domain GC asymptotically follows distributions that are known analytically (chi-squared family), thus facilitating hypothesis testing (Geweke, 1982).

*Note on Redundancy:* A specific argument Hu et al. (2011) make against GC comes from the behaviour of the measure for the system given by their equation 10. Hu et al. (2011) compute the GC from *X*_2_ to *X*_1_ to be zero when the residual η_2_ associated with *X*_2_ has zero variance, but claim that a measure of causal influence should be non-zero in this case. However, in this case, *X*_2_ is a redundant variable, being fully determined by the past of *X*_1_, and therefore, does not influence *X*_1_ once the past of *X*_1_ is taken into account. In other words, *X*_2_ has no independent causal influence on *X*_1_ and it is therefore, entirely consistent for GC to be zero in this case.

GC is not a perfect measure for all stochastic time series: if the true process is not a straightforward multivariate autoregressive process with white-noise residuals, then it becomes only an approximate measure of causal influence. In each real-world scenario, discretion is required in deciding if confounds such as non-linearity and correlations in the noise are mild enough for the measure to remain applicable. In these scenarios, it can be useful to consider a range of different measures such as Phase Slope Index (Nolte et al., 2008), Partial Directed Coherence (Baccalá and Sameshima, 2001), and the Directed Transfer Function (Kaminski and Blinowska, 1991; Kaminski et al., 2001).

In summary, GC measures causal effect in a clear and unambiguous way on stationary multivariate autoregressive processes. We believe that the measure is rightly being widely applied in neuroscience as a measure of directed functional connectivity whenever such models provide a reasonable fit to data. Hu et al.'s (2011) “new causality” compares regression model coefficients rather than prediction errors, and is therefore a measure of causal mechanism. New causality sets out to achieve a different aim from GC, and the divergence of the two measures is not a problem.
